# Fabrication of chitosan/magnetite-graphene oxide composites as a novel bioadsorbent for adsorption and detoxification of Cr(VI) from aqueous solution

**DOI:** 10.1038/s41598-018-33925-7

**Published:** 2018-10-18

**Authors:** Bei Zhang, Runtao Hu, Dejun Sun, Tao Wu, Yujiang Li

**Affiliations:** 1 Key Laboratory of Colloid and Interface Science of Education Ministry, Shandong University, Jinan, 250100 PR China; 2 Shandong Provincial Research Center for Water Pollution Control, School of Environmental Science and Engineering, Shandong University, Jinan, 250100 PR China

## Abstract

By utilizing the synergistic effect of chitosan (CS), magnetite (Fe_3_O_4_) particles, and graphene oxide (GO), a series of efficient and eco-friendly chitosan/magnetite-graphene oxide (CS/MGO) composites were fabricated through a facile chemical route. First, Fe_3_O_4_ particles were chemically deposited on the surface of GO to fabricate MGO hybrid. Then, chitosan was attached on MGO sheets, assembling to CS/MGO composites. According to the results of characterization, the covalent Fe-O-C bonds, electrostatic attraction, and hydrogen bonding between GO, Fe_3_O_4_, and chitosan ensure excellent structural stability and physico-chemical properties. The adsorption of Cr(VI) onto CS/MGO composites was also carried out under various conditions (content of CS, pH, initial concentration, contact time, and temperature). The CS/MGO composites possess high removal capacity for Cr(VI) from aqueous solution. Moreover, results also suggested that the CS/MGO composites had a strong reducing action for Cr(VI). When adsorption occurred, Cr(VI) and Cr(III) were simultaneously removed by CS/MGO composites. In addition, CS/MGO composites could retain good Cr(VI) removal efficiency after reuse over five cycles. CS/MGO composites are expected to have potential applications as easily regenerative bioadsorbents for Cr(VI) polluted water cleanup.

## Introduction

Water pollution by heavy metals constitutes a worldwide environmental concern due to the detrimental effects of many heavy metals on human health and eco-systems. Among all water environmental contaminants, chromium has been known as one of the most toxic substances and chemical elements for centuries^1–3^. Trivalent chromium (Cr(III)) and hexavalent chromium (Cr(VI)) are the two main oxidation states of chromium in aqueous media^4^. Compared with Cr(III), Cr(VI) possesses strong toxicity and carcinogenicity^5^. Cr(VI) is also highly mobile through soil and water in aquatic systems. In addition, Cr(VI) is a strong oxidant and may cause damage once adsorbed by the skin. In contrast, Cr(III) with a low oxidation state is difficult to migrate in aquatic systems due to the formation of precipitate. Cr(III) is also relatively nontoxic. Thus, reduction of Cr(VI) to Cr(III) is significant in the detoxification of Cr(VI)^6,7^. Moreover, Cr(III) should also be removed from aqueous solution to avoid secondary pollution. Simultaneous adsorption and detoxification of Cr(VI) is considered as the most effective, economical and reliable method^8^.

Various adsorbent materials, such as activated carbon^9^, clay minerals^10^, layered double hydroxides^11^, carbon nanotubes^12^, and nanoscale zero-valent iron^13^, have been applied for removing Cr(VI) from aqueous solution. However, traditional adsorbent materials still possess certain problems that limit their practical application. As a result, designing and developing new adsorbent materials to solve these problems is requisite. Recently, bioadsorbents have attracted attention due to their good biocompatibility and biodegradability, strong bioadhesivity and film-forming ability, and excellent reusability^4,14–16^. Natural biomasses may be also employed as modifying agents. Non-harmful and highly effective bioadsorbents are promising adsorbents for the removal of dyes, organic compounds, proteins, and heavy metals^17–20^.

Graphene oxide (GO), one of the most important derivatives of graphene, contains many more polar moieties and oxygen-rich functional groups such as epoxy (C-O-C), hydroxyl (-OH), carboxyl (-COOH), and carbonyl (C=O) groups on its basal planes and at the edges. Taking these oxygen-rich functional groups into account, GO can be not only well dispersed in both water and organic solvents, but also provide more possibility for fabrication of graphene-based multifunctional materials^21–25^. Unfortunately, some disadvantages of GO limit its practical applications, such as that small-sized GO disperses in aqueous solution and forms a stable colloidal suspension, and thus it is challenging to separate and recycle^26,27^. Magnetite (Fe_3_O_4_) particles possess good compatibility, low toxicity, and high magnetic properties, and can be added to as-prepared building blocks (e.g., GO, chitosan) and assemble Fe_3_O_4_-GO (MGO)^28^ and Fe_3_O_4_-chitosan^29^ hybrid. However, MGO sheets tend to restack together and form laminated micro-structures. To prevent the aggregation of MGO sheets and augment MGO dispersity in water, one of the most efficient methods is to complex MGO with other functional materials^28–30^. Chitosan (CS) is a cationic biopolymer obtained from chitin via a deacetylation process, whereby the acetamide groups are hydrolyzed to produce acetate ions and amino (-NH_2_) groups. In the acidic condition, the amino groups of CS are easily pronated and can bind anionic functional groups or anions. The presence of large amounts of amino groups on the CS backbone plays a key role in the hybridization process. According to the above-mentioned processes, MGO sheets can be easily bound to CS by mutual reactions between oxygen-containing functional groups on MGO and amino groups on CS. Integrating MGO with CS achieves a versatile bioadsorbent because of its advantages, including good biocompatibility and biodegralability, high adsorption capacity, and excellent separation properties^31–33^. And the CS/MSO composites possess excellent properties for removal of dyes^32^ and heavy metal^31,33^.

The aims of the present work are: (1) to fabricate CS/MGO composites and characterize them by HRTEM, FE-SEM, XRD, VSM, FT-IR, and XPS; (2) to investigate the effects of mass ratio of CS to MGO, pH, initial concentration, contact time, and temperature on Cr(VI) removal; (3) to explore and discuss the superiority of CS/MGO composites concerning adsorption aspects and removal mechanism; and (4) to investigate the reusability of the CS/MGO composites. Compared with the extant literature on CS/MGO^34^, we modified the synthesis process to obtain a series of composites with different mass ratio of CS to MGO, so that the structural features of composites could be adjusted and controlled to achieve an excellent removal property for pollutants. In addition, concerning not only adsorption but also detoxification of Cr(VI), we performed a characterization and batch experiment to investigate the chemical transformation of Cr(VI) to Cr(III) and adsorption-detoxification mechanism by CS/MGO composites. Thus, our work may provide a novel understanding of CS/MGO with heavy metals.

## Results

### Characterization

**Figure 1:**
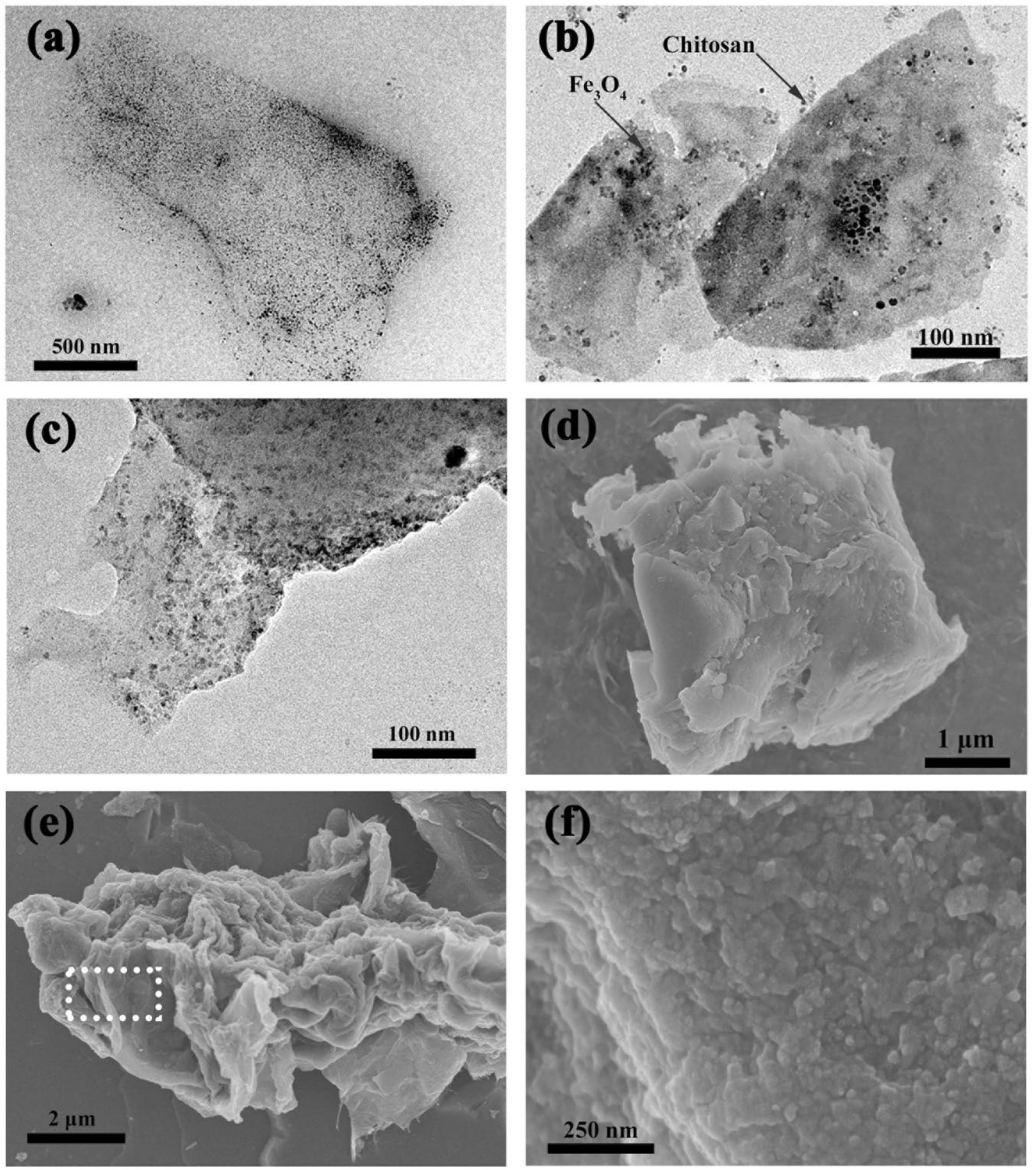
HRTEM images (**a**–**c**) and FE-SEM images (**d**–**f**) of MGO (**a**,**d**) and CS/MGO composites (**b**,**c**,**e**,**f**). The region enclosed by a white box (**e**) is enlarged and shows a close view of the nanoparticles (**f**).

Figure 1 shows HRTEM and FE-SEM images of MGO and CS/MGO composites. A few graphene oxide sheets constitute the main framework (Fig. 1a,b,d,e), and the magnetic nanoparticles are tightly anchored onto the lamellas (Fig. 1c,f). Compared with MGO composites (Fig. 1a), the GO sheets in CS/MGO composites (Fig. 1b) exhibit some overlapping and agminate, indicating that chitosan can play the role of a bridge between the lamellas. Furthermore, the FE-SEM image of CS/MGO composites (Fig. 1e) exhibits more obvious folding and wrinkling than MGO composites (Fig. 1d) due to the existence of chitosan. Meanwhile, the active sites of chitosan are adequately exposed between GO sheets, which can promote the interaction of CS/MGO composites with target pollutants.

**Figure 2:**
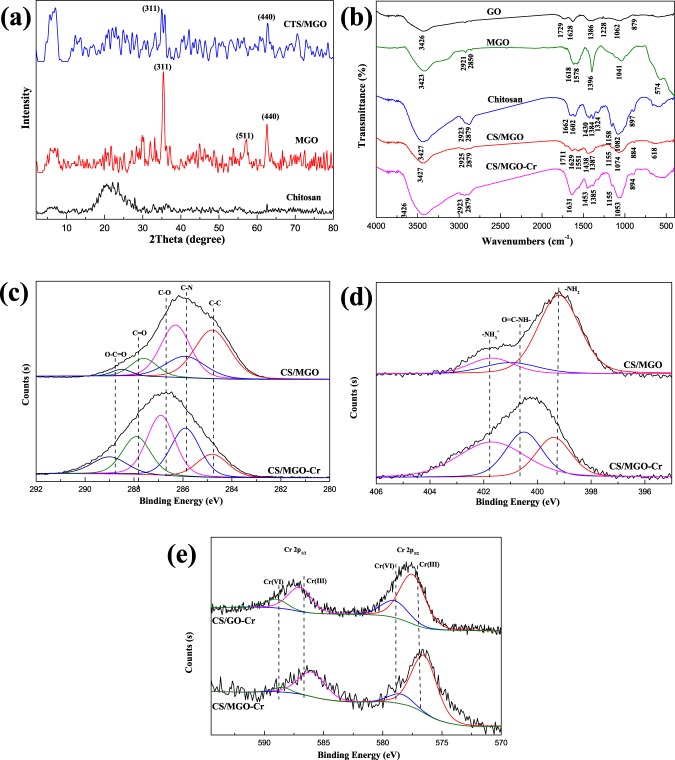
XRD patterns (**a**) of chitosan, MGO, and CS/MGO; FT-IR spectra (**b**) of GO, MGO, chitosan, CS/MGO, and CS/MGO-Cr; XPS spectra: C 1 s spectra (**c**), N 1 s spectra (**d**) of CS/MGO and CS/MGO-Cr, and Cr 2p spectra (**e**) of CS/GO-Cr and CS/MGO-Cr.

The XRD patterns of GO (Supplementary Fig. S1a), chitosan, MGO, and CS/MGO (Fig. 2a) reveal the phase transformation before and after composition. The diffraction peak at 2θ = 11.18°, which corresponds to the typical (001) crystal plane of GO, disappears in the CS/MGO composites. In addition, the broad peak around 20° in chitosan becomes weak in the CS/MGO composites^8^. The results show that the phase structure of GO and chitosan in CS/MGO composites becomes amorphous due to hybridization in between them^35^. Moreover, the diffraction peaks at 2θ = 34.94°, 60.68°, and 62.78° in MGO can be assigned to (311), (511), and (440) crystal planes of Fe_3_O_4_, respectively. Correspondingly, the weak peaks at 2θ = 35.48° and 62.14° of the (311) and (440) plane in CS/MGO composites indicate the existence of Fe_3_O_4_ nanoparticles after composition^36^.

The magnetic properties of samples are measured with VSM characterization. According to the magnetization curves (Supplementary Fig. S1b), the magnetic remanence (Mr) and coercivity (Hc) values are 0.04 emu/g and 1.63 Oe for MGO and 0.02 emu/g and 1.60 Oe for CS/MGO composites, respectively, revealing that both composites exhibit superparamagnetic behavior due to the existence of Fe_3_O_4_ nanoparticles. For CS/MGO composites, the saturation magnetization (Ms, 2.52 emu/g) is smaller than the value of MGO (5.03 emu/g), which is due to the difference of composition between these samples^3,36^. Because only the Fe_3_O_4_ nanoparticles in samples possess a good magnetic response, the relatively low amount of magnetic nanoparticles in CS/MGO (chitosan: GO: Fe_3_O_4_ = 50 wt%: 25 wt%: 25 wt%) compared to MGO (GO: Fe_3_O_4_ = 50 wt%: 50 wt%) result in the small saturation magnetization for CS/MGO. The photographs of magnetic separation show that CS/MGO composites can be effectively separated from aqueous solution.

FT-IR spectra are performed to investigate the functional groups transformation of CS/MGO composites. As shown in Fig. 2b, the bands of GO at 3426, 1729, 1628, and 1062 cm^−1^ are assigned to the stretching vibrations of –OH, C=O, C=C, and C-OH, respectively^36,37^. The vibrational bands at 1228 and 837 cm^−1^, which are assigned to the anti-symmetric and symmetric stretching vibrations of C-O-C, respectively, disappear in MGO composites due to the formation of covalent bonds between the oxygen-containing functional groups of GO and Fe atoms of Fe_3_O_4_^37^. Two additional vibrational bands at 2921 and 2850 cm^−1^ can be attributed to the C-H anti-symmetric and symmetric stretching vibration, respectively, indicating that a partial sp2-hybridized carbon plane has been recovered. In the spectrum of chitosan, the bands at 1662 and 1602 cm^−1^ are characteristics of the amide I stretching vibration of -NHCO- and N-H bending of –NH_2_, respectively^22,36^. Concerning CS/MGO composites, both the characteristic bands of MGO and chitosan appear in the spectrum, revealing that main functional groups are reserved to interact with Cr(VI). It should be noted that the vibrations of amide I, N-H, C=O, and C-OH have weakened, which is ascribed to electrostatic interaction and the formation of covalent bonds between the oxygen-containing functional groups of MGO and chitosan. After the adsorption of Cr(VI), the bands of CS/MGO at 1711 and 1551 cm^−1^, which correspond to amide I and C=O, respectively, disappear in the spectrum of CS/MGO-Cr, indicating that the nitrogen-containing and oxygen-containing functional groups are involved in the removal of Cr(VI).

To further verify the results obtained from the FT-IR analysis, XPS spectra and quantitative XPS analysis results of CS/MGO, CS/MGO-Cr, CS/GO-Cr, and chitosan-Cr are presented in Fig. 2c,d,e, Supplementary Fig. S2, Supplementary Tables S1 and S2, respectively. In the high-resolution XPS C1s spectra of CS/MGO and CS/MGO-Cr (Fig. 2c), the raw peaks can be deconvoluted into the fitting peaks at 284.8 (C-C/C=C), 285.9 (C-N), 286.3 (C-O), 287.6 (C=O), and 288.5 (O-C=O) eV^4,36^. After Cr adsorption, the relative content of the C-C is significantly reduced from 36.40% to 13.13%, which can be attributed to the formation of CS/MGO-Cr complex, in which the rich electrons in the GO frameworks are donated to Cr(VI) ions. Consequently, more C-N, C=O, and O-C=O are observed in higher binding energy peaks due to the combination between carbon atoms and nitrogen and oxygen atoms with long pair electrons^38^. As shown in Fig. 2d, the N 1s XPS spectra can be deconvoluted into three peaks with binding energies at 399.2, 401.0, and 401.7 eV, attributed to amine (-NH_2_), amide (O=C-NH-), and protonated amine (-NH_3_^+^), respectively^4,22,39^. In comparison with pristine CS/MGO composites, the content of amine decreases from 71.54% to 25.72% after Cr adsorption. In addition, many nitrogen atoms exist in a more oxidized state on the surface of CS/MGO composites. Moreover, the increase in protonated amine reveals the existence of electrostatic interaction in the adsorption process^22^. To further examine the chemical transformation of Cr(VI) adsorbed on the different samples, the Cr 2p XPS spectra of chitosan-Cr, CS/GO-Cr, and CS/MGO-Cr are deconvoluted into four peaks (Fig. 2e, Supplementary Fig. S2). The bands at 577.5 and 587.0 eV can be attributed to Cr (III), indicating that Cr(VI) is partly reduced during the adsorption process^2,40^. Especially, CS/MGO composites possess a higher content of Cr(III) than chitosan and CS/GO, as shown in Table S2. According to the data of Cr 2p_3/2_, 86.74% of Cr(VI) adsorbed on CS/MGO composites is reduced to Cr(III), which is higher than chitosan (21.71%) and chitosan/graphene oxide composites (73.63%). This result is due to that the recovered sp2-hybridized carbon plane in MGO promotes the charge transfer and augments the reduction of Cr(VI) as excellent electron donors^40–43^. Meanwhile, the oxygen-containing and nitrogen-containing groups in GO sheets and chitosan are involved in the reduction of Cr(VI). Fe_3_O_4_ nanoparticles bonding with oxygen-containing groups in GO sheets can also enhance reduction capacity via promoting adsorption and charge transfer between CS/MGO and Cr(VI)^44,45^.

### Adsorption and detoxification of Cr(VI) on CS/MGO composites

**Figure 3:**
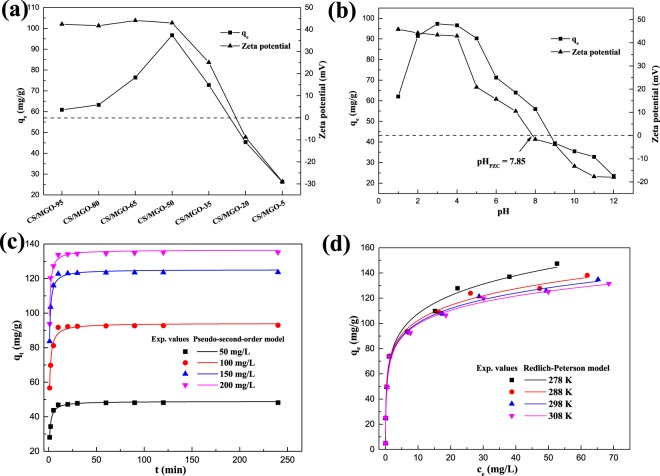
Effect of chitosan content in CS/MGO composites on equilibrium adsorption capacities (q_e_) for Cr(VI) and zeta potential in aqueous solution (**a**) effect of initial pH on equilibrium adsorption capacities (q_e_) for Cr(VI) and zeta potential of CS/MGO composites in aqueous solution (**b**) adsorption kinetics of Cr(VI) on CS/MGO composites and fitting of pseudo-second-order kinetic model to the experimental values (**c**) adsorption isotherm of Cr(VI) on CS/MGO composites and fitting of the Redlich-Peterson model to the experimental values (**d**). Conventional experimental conditions: initial pH = 4, initial concentration = 100 mg/L, adsorbent dosage = 1.0 g/L, contact time = 4 h, and T = 298 K.

The adsorption and detoxification characteristics of CS/MGO composites for the removal of Cr(VI) in aqueous solution are examined by batch experiments. Based on the investigation above, chitosan in CS/MGO composites plays the main role in attracting Cr(VI) anion in aqueous solution with protonated amine. Thus, the effect of chitosan contents in CS/MGO composites on equilibrium adsorption capacities (q_e_) for Cr(VI) and zeta potential in aqueous solution is firstly studied. The zeta potential contributes to understanding the charge interaction between adsorbents and pollutants^46^. As shown in Fig. 3a, the value of the zeta potential of CS/MGO composites maintains + 40 mV when the chitosan contents are above 50%, and then gradually reduces with decreasing chitosan contents. However, the adsorption capacities at high chitosan contents (>50%) are lower than CS/MGO-50 in spite of the same zeta potential and more adsorption sites in chitosan. This is because Cr(VI)-adsorbed chitosan is dispersion-stable and difficult to separate from solution at high chitosan contents. For CS/MGO-50, abundant GO sheets anchored Fe_3_O_4_ nanoparticles function as a support platform that can promote the adsorption and separation capacity of chitosan. When the chitosan content is above 50%, the adsorption capacities present a similar trend to zeta potential due to subdued affinity. It should be noted that CS/MGO-5 still possesses 26.26 mg/g of adsorption capacity with the negative value of zeta potential, while electrostatic attraction is suppressed due to deprotonation under the alkaline condition. The multiple interactions between the negatively charged surface of CS/MGO composites and Cr(VI) anions need to be further elucidated.

The effect of pH is a key factor for managing the adsorption process and investigating the adsorption mechanism. As shown in Fig. 3b, equilibrium adsorption capacities (q_e_) increase at pH 1–3, and then gradually decrease at pH 4–12. At pH < 3, H_2_CrO_4_ and HCrO_4_^−^ are the dominant species of Cr(VI), and protonated Cr(VI) anions possess low affinity to CS/MGO composites. In addition, the partial dissolution and poor combination stability of CS/MGO composites weaken the attraction for Cr(VI), leading to low adsorption capacity at pH < 3. With increasing pH, the adsorption efficiency decreases at pH > 4. The zeta potential of the composites plays an important role in the adsorption process. As shown in Fig. 3b, CS/MGO composites possess a positively charged surface with pH of point of zero charge (pH_PZC_) = 7.85. At pH < pH_PZC_, the favorable adsorption to Cr(VI) anions by strong electrostatic attraction^47^ ensures higher adsorption efficiency than at pH > pH_PZC_. Moreover, as shown in Supplementary Table S3, the final pH increases after the adsorption of Cr(VI) at pH < pH_PZC_, which is ascribed to protonic and electronic consumption during the adsorption and reduction of Cr(VI) by CS/MGO composites^48^. The reduction process is given as the following reactions. It should be noted that at pH > pH_PZC_, CS/MGO composites can adsorb a certain amount of Cr(VI) in spite of the electrostatic repulsion. The chelation of chitosan and surface complexation of Fe_3_O_4_ nanoparticles in CS/MGO composites with Cr(VI) play a major role in the alkaline condition. Meanwhile, the final pH reduces slightly at pH > pH_PZC_ due to deprotonation of CS/MGO composites.

1 $${\rm{HCr}}{{{O}}_{4}}^{-}+7\,{H}^{+}+3\,{e}^{-}\to C{r}^{3+}+4\,{H}_{2}{O}$$

2 $${{\rm{Cr}}}_{2}{{O}_{7}}^{2-}+14\,{{H}}^{+}+6\,{e}^{-}\to 2\,C{r}^{3+}+7\,{H}_{2}{O}$$

Adsorption kinetics is then performed to investigate the adsorption process. The effects of contact time and initial concentration on the adsorption of Cr(VI) are presented in Fig. 3c. The adsorption equilibrium time of CS/MGO composites for each initial concentration of Cr(VI) is less than 15 min, revealing that the adsorption process is relatively rapid due to the adequate attraction. To further study the adsorption process, nonlinear pseudo-second-order (Fig. 3c), nonlinear pseudo-first-order (Supplementary Fig. S3a), intra-particle diffusion (Supplementary Fig. S3b), and Boyd (Supplementary Fig. S3c) kinetic models are adopted. The modeling results are listed in Table 1. The kinetic rate equations can be written as^11,49,50^:

3 $${{\rm{q}}}_{{\rm{t}}}={{\rm{q}}}_{{\rm{e}}}({\rm{1}}-{{\rm{e}}}^{-{{\rm{k}}}_{1}{\rm{t}}})$$

4 $${{\rm{q}}}_{{\rm{t}}}=\frac{{{\rm{k}}}_{2}{{{\rm{q}}}_{{\rm{e}}}}^{2}{\rm{t}}}{{\rm{1}}+{{\rm{k}}}_{2}{{\rm{q}}}_{{\rm{e}}}{\rm{t}}}$$

5 $${{\rm{q}}}_{{\rm{t}}}={{{\rm{k}}}_{{\rm{p}}}}^{{{\rm{t}}}^{\frac{1}{2}}}+{\rm{I}}$$

6 $${{\rm{B}}}_{{\rm{t}}}=-{\rm{0.4977}}-\,\mathrm{ln}(1-\frac{{{\rm{q}}}_{{\rm{t}}}}{{{\rm{q}}}_{{\rm{e}}}})$$

where q_e_ and q_t_ correspond to the amount of Cr(VI) adsorbed per unit mass of CS/MGO (mg/g) at equilibrium and at time t, and k_1_ (min^−1^) and k_2_ (g·mg^−1^ min^−1^) are the rate constant for pseudo-first-order and pseudo-second-order. k_p_ (mg·g^−1^·min^0.5^) and I are the rate constant and intercept of intra-particle diffusion; and B_t_ is a mathematical function of the fraction of adsorbed Cr(VI) at time t.

**Table 1:** Pseudo-first-order, pseudo-second-order, intra-particle diffusion, and Boyd model kinetic parameters for the adsorption of Cr(VI) onto CS/MGO composites at 298 K.

C_0_ (mg/L)	Pseudo-first-order			Pseudo-second-order			Intra-particle diffusion				Boyd model		
	q_e1,cal_ (mg/g)	k_1_ (min^−1^)	R^2^	q_e2,cal_ (mg/g)	k_2_ × 10^2^ (g·mg^−1^ min^−1^)	R^2^	k_p1_ (mg·g^−1^·min^0.5^)	R^2^	k_p2_ (mg·g^−1^·min^0.5^)	R^2^	slope	intercept	R^2^
50	47.39	0.75	0.9376	48.93	2.73	0.9856	12.48	0.9898	0.10	0.5773	0.03	2.39	0.7002
100	91.21	0.83	0.8987	94.11	1.59	0.9844	19.01	0.8972	0.09	0.8122	0.03	2.40	0.6836
150	122.23	1.07	0.9497	125.18	1.74	0.9828	24.59	0.8002	0.07	0.8133	0.02	2.99	0.5551
200	133.68	1.19	0.9664	136.55	1.89	0.9505	24.41	0.5147	0.11	0.8743	0.02	2.82	0.6379

According to the values of R^2^ at different initial concentrations of Cr(VI), the adsorption kinetics are better fitted by the pseudo-second-order model, indicating that the adsorption process is controlled by multiple factors. Furthermore, two-stage kinetic fitting on intra-particle diffusion reveals that the intra-particle diffusion is the rate-determining step at the first stage when adsorption sites are abundant. At the second stage, the rate of surface reactions (physical and chemical adsorption) may restrict the adsorption process. In addition, the Boyd model is applied to investigate pore and film diffusion in the adsorption process^50^. The results of fitting reveal that film diffusion constitutes the key factor in the adsorption process, which is also related to the surface reactions.

Figure 3d and Supplementary Fig. S4 present the adsorption isotherms of Cr(VI) on CS/MGO composites at different temperatures. The parameters of adsorption isotherms by Langmuir, Freundlich, and Redlich-Peterson models are presented in Table 2. The equations are expressed as follows^51–53^:

**Table 2:** The parameters of Langmuir, Freundlich, and Redlich-Peterson models for the adsorption of Cr(VI) onto CS/MGO composites.

Temperature (K)	Langmuir isotherm			Freundlich isotherm			Redlich-Peterson model			
	q_max_ (mg/g)	K_L_ (L/mg)	R^2^	K_F_	n	R^2^	K_RP_ (L/g)	a_RP_ L/mg	β	R^2^
278	129.38	1.46	0.9236	59.12	4.71	0.9610	550.65	7.38	0.83	0.9872
288	123.31	1.38	0.9160	58.04	4.62	0.9479	849.54	11.65	0.85	0.9702
298	122.03	1.23	0.9300	56.52	4.52	0.9413	535.16	7.27	0.86	0.9734
308	120.97	1.11	0.9336	54.42	4.14	0.9426	363.63	4.81	0.87	0.9788

Langmuir isotherm:

7 $${{\rm{q}}}_{{\rm{e}}}=\frac{{{\rm{q}}}_{{\rm{\max }}}{{\rm{K}}}_{{\rm{L}}}{{\rm{c}}}_{{\rm{e}}}}{(1+{{\rm{K}}}_{{\rm{L}}}{{\rm{c}}}_{{\rm{e}}})}$$

Freundlich isotherm:

8 $${{\rm{q}}}_{{\rm{e}}}={{\rm{K}}}_{{\rm{F}}}{{{\rm{c}}}_{{\rm{e}}}}^{1/{\rm{n}}}$$

Redlich-Peterson model:

9 $${{\rm{q}}}_{{\rm{e}}}=\frac{{{\rm{K}}}_{{\rm{RP}}}{{\rm{c}}}_{{\rm{e}}}}{{\rm{1}}+{{\rm{a}}}_{{\rm{RP}}}{{{\rm{c}}}_{{\rm{e}}}}^{{\rm{\beta }}}}$$

where q_e_ is the amount of Cr(VI) adsorbed at equilibrium (mg/g); c_e_ is the concentration of Cr(VI) at equilibrium (mg/L); q_max_ is the adsorption capacity when the adsorbent is fully covered (mg/g); K_L_ is the Langmuir adsorption constant (L/mg); K_F_ is the Freundlich isotherm constant; n is the heterogeneity factor; K_RP_ (L/g), a_RP_ (L/mg), and β are the empirical coefficients of the Redlich-Peterson model; and the value of β lies between 0 and 1.

According to the simulated parameters (Table 2), the Redlich-Peterson model possesses the best fitting with high values of R^2^ compared to the Langmuir and Freundlich models. It should be noted that the values of β for the Redlich-Peterson model are higher than 0.83. Considering that the Redlich-Peterson model is derived from a combination of two relations approaching Freundlich and Langmuir, the high values of β indicate that the adsorption process is heterogeneous^51,52^. In addition, the Freundlich model provides a better fitting than the Langmuir. The protonated amine, oxygen-containing functional groups, and Fe_3_O_4_ nanoparticles are involved in the heterogeneous adsorption. The values of K_L_, K_F_, and n reflect adsorption capacity and affinity, which decrease with increasing temperature. Compared with other adsorbents^3,16^, the CS/MGO composites possess desirable adsorption properties with abundant sites. The thermodynamic parameters are calculated using the following equations^29,30,54^:

10 $${{\rm{K}}}_{{\rm{C}}}=\frac{{{\rm{q}}}_{{\rm{e}}}}{{{\rm{c}}}_{{\rm{e}}}}$$

11 $${\rm{\Delta }}{\rm{G}}^\circ =-{\rm{RT}}\,\mathrm{ln}\,{{\rm{K}}}_{{\rm{C}}}$$

12 $$\mathrm{ln}\,{{\rm{K}}}_{{\rm{C}}}=-\frac{{\rm{\Delta }}{\rm{H}}^\circ }{{\rm{RT}}}+\frac{{\rm{\Delta }}{\rm{S}}^\circ }{{\rm{R}}}$$

where K_C_ is the equilibrium constant; q_e_ and c_e_ are the same as for the isotherm equations (initial concentration = 100 mg/L); T is the temperature in Kelvin; and R is the gas constant (8.314 J·mol^−1^·K^−1^).

As shown in Table 3, the negative values of ΔG° suggest that the process is spontaneous. In addition, the values increase with rising temperature due to the promotion of adsorption at high temperatures. The negative value of ΔH° indicates the exothermic nature of the adsorption. Moreover, the interface between the CS/MGO composites and Cr(VI) solution becomes active and random with the positive value of ΔS°.

**Table 3:** Thermodynamic parameters for the adsorption of Cr(VI) onto CS/MGO composites.

Temperature (K)	ΔG° (KJ·mol^−1^)	ΔH° (KJ·mol^−1^)	ΔS° (J·mol^−1^·K^−1^)
278	−6.12	−3.58	9.14
288	−6.21		
398	−6.31		
308	−6.40		

### Structural stability and reusability experiment

**Figure 4:**
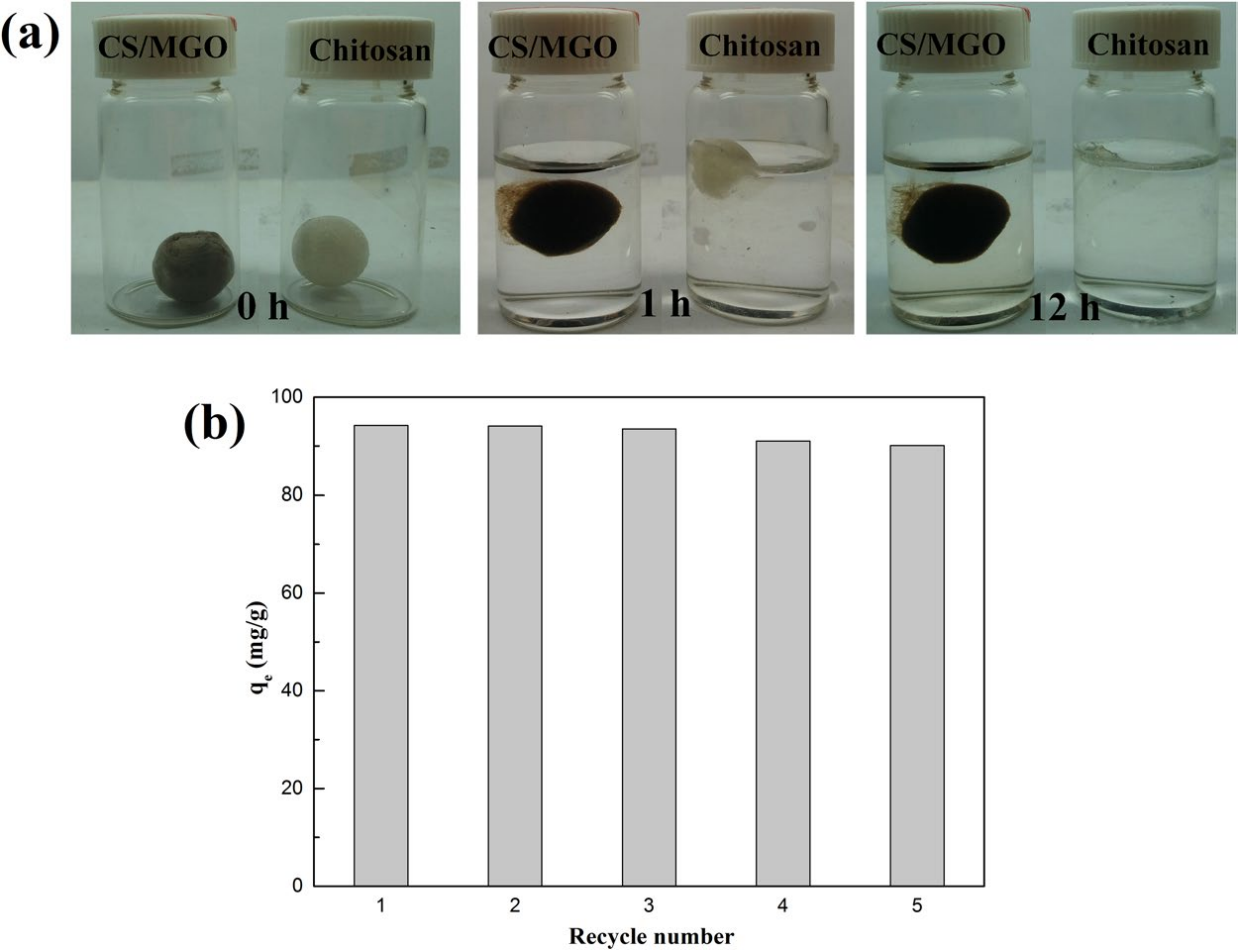
The morphologic changes of CS/MGO and chitosan beads with time, being dipped in aqueous solution at pH 4 (**a**) recycling of CS/MGO composites in the adsorption of Cr(VI) (**b**).

To elucidate the structural strength and wet-state stability of the samples, morphologic changes are recorded, as shown in Fig. 4a. The CS/MGO bead maintains its original shape with slight swelling after 12 h. In contrast, the chitosan bead undergoes an obvious change after 1 h, and almost completely disappears due to dissolution. In addition, the regeneration capability of CS/MGO composites is evaluated, as shown in Fig. 4b. The CS/MGO composites retain high adsorption capacities (q_e_ > 90 mg/g) after five recycles, revealing that CS/MGO composites possess excellent regenerability and adsorptive properties. These results demonstrate that CS/MGO, with better structural stability and reusability, possesses high potential for practical application.

## Discussion

**Figure 5:**
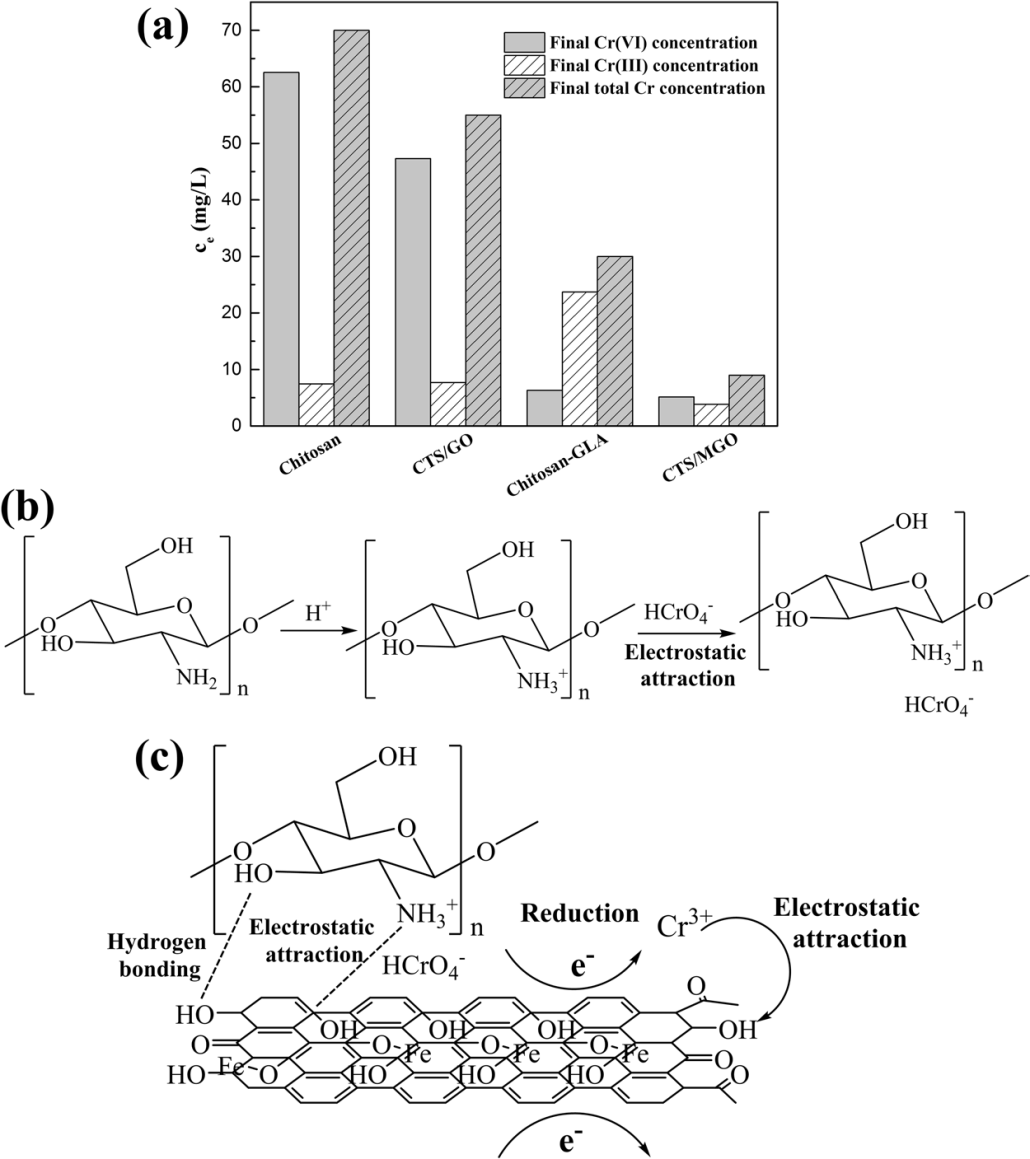
Comparison of chromium species (Cr(VI), Cr(III), and total Cr) equilibrium concentration by chitosan, CS/MGO, chitosan-GLA, and CS/MGO (**a**) schematic of electrostatic attraction (**b**) and reduction mechanism (**c**) of Cr(VI) onto CS/MGO composites.

Based on the characterization and adsorption experiment above, the relationship between structure and adsorption-detoxification properties is further clarified. A comparison of chromium species^55^ (Cr(VI), Cr(III), and total Cr) equilibrium concentration by chitosan, CS/MGO, chitosan-GLA, and CS/MGO (Fig. 5a) is also carried out to elucidate this relationship.

First, electrostatic attraction plays the main role of the driving force for the adsorption of Cr(VI) in aqueous solution. The positively-charged surface of chitosan possesses strong electrostatic attraction for Cr(VI) anions (Fig. 5b). Although pristine chitosan has relatively low adsorption capacity, glutaraldehyde cross-linked chitosan (chitosan-GLA) possesses high capacity for Cr(VI) due to electrostatic attraction (Fig. 5a). Moreover, the results of XPS analysis demonstrate that protonated amine increases with the adsorption process, enhancing electrostatic attraction. In contrast, the existence of electrostatic repulsion between oxygen-containing functional groups in GO sheets and Cr(VI) anions is distant for adsorption, leading to the lower adsorption capacities of CS/GO than CS-GLA (Fig. 5a). After anchoring magnetic nanoparticles, GO sheets in MGO are partly reduced according to FT-IR spectra, which weakens electrostatic repulsion. Thus, with abundant chitosan combining on the MGO sheets, the CS/MGO composites can efficiently adsorb Cr(VI) anions and rapidly achieve adsorption equilibrium.

GO sheets in CS/MGO composites then enhance adsorption-detoxification capacity. Recovery of the sp2-hybridized carbon plane in MGO can promote charge transfer of surface reactions as electron donors in the adsorption process (Fig. 5c), thus enhancing the reduction capacity of CS/MGO composites. After the adsorption of Cr(VI), the oxidized state carbon atoms increase with decreasing C-C from XPS analysis. Accordingly, there are more Cr(III) reduced from Cr(VI) for CS/MGO composites than chitosan and CS/GO. Moreover, in comparison with CS/MGO composites (Fig. 5a), chitosan-GLA has more residue of Cr(III), which can cause secondary pollution of Cr. Conversely, GO sheets in CS/MGO composites enhance the adsorption of Cr(III) cation with residual oxygen-containing functional groups by electrostatic attraction.

Fe_3_O_4_ nanoparticles augment adsorption-detoxification and separation capacities. Pristine Fe_3_O_4_ nanoparticles possess strong adsorption capacity for Cr(VI) via surface complexation. After the Fe atoms combine with oxygen-containing functional groups in GO sheets by Fe-O-C bonds, Fe_3_O_4_ nanoparticles can also promote charge transfer with Fe(II) and Fe(III)^56^. In addition, Fe_3_O_4_ nanoparticles anchored onto GO sheets ensure efficient magnetic separation, thus improving reusability and practical application.

In summary, CS/MGO composites are successfully synthesized for the adsorption-detoxification of Cr(VI) in aqueous solution. The nitrogen-containing functional groups of chitosan in CS/MGO composites play the main role of adsorption and detoxification. GO sheets, anchored with Fe_3_O_4_ nanoparticles, provide chitosan with abundant attachment sites for Cr(III) and Cr(VI), thus enhancing structural strength and adsorption capacity. Meanwhile, the sp2-hybridized carbon atom layer of MGO promotes the charge transfer of surface reactions and reduction properties for Cr(VI) as electron donors. The chemical shift of Cr revealed that 86.74% of Cr(VI) adsorbed on CTS/MGO composites was reduced to Cr(III), which was higher than chitosan (21.71%) and chitosan/graphene oxide composites (73.63%). Thus, CS/MGO composites possess excellent adsorption capacities, detoxification properties, structural stability, and regeneration and magnetic separation capability, which can be applied to rapidly remove and separate Cr(VI) in aqueous solution.

## Methods

### Chemicals and materials

Graphite powder (granularity ≤30 μm) was purchased from the Sinopharm Chemical Reagent Co., Ltd., with high chemical purity. KMnO_4_, FeCl_3_·6H_2_O, FeSO_4_·7H_2_O, H_2_SO_4_ (98%), H_2_O_2_ (30%), and NH_3_·H_2_O of analytical grade were purchased from the Laiyang Institute of Chemical Reagents. Chitosan with 85% deacetylation was obtained from the Sinopharm Chemical Reagent Co., Ltd. The chemical structures of chitin and chitosan are shown in Supplementary Fig. S5. Glutaraldehyde (50%) was used as the cross-linking agent, which was purchased from the Tianjin Damao Chemical Reagent Co., Ltd. Potassium dichromate (K_2_Cr_2_O_7_) was provided by the Sinopharm Chemical Reagent Co., Ltd. All other reagents used were analytical grade without further purification. Deionized (DI) water was produced using an ultra-pure water purifier system, with a resistivity of 18.2 MΩ·cm^−1^.

### Synthesis of graphene oxide and magnetic graphene oxide

Graphene oxide (GO) was synthesized using an improved Hummers method^57,58^. The GO dispersion was obtained by ultrasonication at room temperature. Then, the magnetite-graphene oxide (MGO) was further synthesized by co-precipitating iron salts onto GO sheets in basic solution. The detailed procedure for GO and MGO synthesis is provided in the Supporting Information.

### Synthesis of chitosan/magnetite-graphene oxide composites

The synthetic process of chitosan/magnetite-graphene oxide (CS/MGO) composites is described as follows^31,32^. Chitosan (1.0 g) was dissolved in 100 mL of acetic solution (1% v/v), and the mixture was continually stirred at 30 °C for 24 h. MGO dispersion (500 mL, 2.0 g/L) was then added into the mixture, and stirring was continued at 60 °C for 30 min. Next, 10 mL of glutaraldehyde (GLA, 50%) was added with stirring at 60 °C for another 2 h. The resulting precipitate was magnetically separated, and CS/MGO dispersion was obtained by washing thoroughly with DI water. The final product, CS/MGO composites, was obtained by a freeze-drying process. The amount of chitosan in CS/MGO could be controlled by changing the dosage of chitosan. A series of MGO with different chitosan contents of 95, 80, 65, 50, 35, 20, and 5% were referred to as CS/MGO-95, CS/MGO-80, CS/MGO-65, CS/MGO-50, CS/MGO-35, CS/MGO-20, and CS/MGO-5, respectively. The product CS/MGO-50 was used as the main CS/MGO composites in the characterization and adsorption experiments.

Chitosan/graphene oxide (CS/GO) was also prepared in a similar method, except that no magnetic iron oxide nanoparticles were added. In addition, glutaraldehyde cross-linked chitosan (chitosan-GLA) was obtained without adding MGO.

### Synthesis of microsphere samples

To investigate the structural stability of samples in aqueous solution, chitosan, MGO, and CS/MGO beads were prepared by a freeze-drying process with spherical molds (diameter = 10.0 mm). The dispersion of samples (10.0 g/L) was achieved by stirring at 30 °C for 6 h. Then, the mixture was respectively added into the spherical molds. The chitosan and CS/MGO beads were successfully obtained via a freeze-drying process. The products of MGO were powders with a fragile structure. Photographs of samples are presented in Supplementary Fig. S6.

### Characterization

The morphology of CS/MGO composites was characterized by high-resolution transmission electron microscope (HRTEM, JEM-2100, Japan) and field-emission scanning electron microscopy (FE-SEM, Hitachi SU8010, Japan). X-ray diffraction (XRD) spectra were measured to examine the structural properties of composites on a diffractometer (Rigaku D-Max 2200, Japan). The magnetization curves of the samples were obtained to confirm the magnetic separation capability with a vibrating sample magnetometer (VSM, LDJ 9500, U.S.A.). To study the structural transformation of samples before and after composition and adsorption of Cr(VI), Fourier Transform Infrared spectroscopy (FT-IR) spectra were obtained using a FT-IR spectrophotometer (JASCO, FT/IR-6300, Japan). X-ray photoelectron spectroscopy (XPS) analysis was further carried out with an X-ray photoelectron spectrometer (Thermo ESCALAB 250XI, U.S.A.). The zeta potential of the samples was determined by a zeta-potential analyzer (ZetaPALS, Brookhaven, U.S.A.).

### Adsorption experiment

To investigate the adsorption performance of Cr(VI) on CS/MGO composites, an adsorption experiment was performed using batch techniques. The conventional experimental conditions are described as follows. The adsorbents (1.0 g/L) were added into 100 mg/L Cr(VI) solution at initial pH 4. After shaking (150 rpm) at 298 K for 4 h, the supernatants of the mixture were separated with a magnet next to the container bottle to determine the concentration of Cr(VI).

The experimental conditions were also separately changed to investigate the effect of different factors. Specifically, the effect of chitosan content in CS/MGO composites was determined by changing the content from 95% to 5% during synthesis and adsorption. For the effect of initial pH, the initial solution pH (1–12) was adjusted by NaOH and HCl aqueous solution. For kinetic analysis, Cr(VI) solution with different initial concentrations (50, 100, 150, and 200 mg/L) was used at contact time ranging from 1 to 240 min. For isotherm investigation, Cr(VI) solution with an initial concentration ranging from 5 to 200 mg/L was used at different temperatures (278, 288, 298, and 308 K).

The concentration of Cr(VI) and total Cr was measured by the standard diphenylcarbazide method. For Cr(VI), the red-violet product, generated from Cr(VI) and diphenylcarbazide under an acid condition, was measured using an UV/vis spectrometer (TU-1810 PC, China) at 540 nm^59,60^. For total Cr, the solution was firstly reacted with KMnO_4_ under an acid condition to ensure total oxidation of Cr(III). Then, the process for determination of total Cr was the same as the measure of Cr(VI). Finally, the concentration of Cr(III) was calculated as the difference value of total Cr and Cr(VI) (total Cr = Cr(VI) + Cr(III)). The Cr-adsorbed samples (CS/MGO-Cr, CS/MGO-Cr, and chitosan-Cr) were collected for the characterization of FT-IR and XPS.

### Structural stability and reusability experiment

For the structural stability experiment of the samples in aqueous solution, chitosan beads and CS/MGO beads were severally added into a 30-mL glass bottle with 20 mL DI water. The pH of the aqueous solution was adjusted to 4. The bottles were then placed in a shaking incubator with 150 rpm at 25 °C for 12 h. The structural changes of the samples were recorded for analysis.

Desorption of adsorbed-Cr(VI) was performed in the NaOH aqueous solution (0.1 mol/L). The regenerated adsorbents were collected by magnetic separation, and then reused in the next cycle of the adsorption experiment at pH 4. The adsorption-desorption experiments were conducted for five cycles.

## Electronic supplementary material

Supplementary Information
