# Supplementary material for: Fabrication of chitosan/magnetite-graphene oxide composites as a novel bioadsorbent for adsorption and detoxification of Cr(VI) from aqueous solution
Source: Sci Rep. 2018 Oct 18;8:15397. doi: 10.1038/s41598-018-33925-7 (PMC6193940; doi:10.1038/s41598-018-33925-7)
Supplement: Supplementary file 1 — Supplementary Information [file 41598_2018_33925_MOESM1_ESM.pdf]

Supporting Information

Fabrication of chitosan/magnetite-graphene oxide composites as a novel bioadsorbent for adsorption and detoxification of Cr(VI) from aqueous solution

Bei Zhang [a, b], Runtao Hu [a], Dejun Sun [a], Tao Wu [a,*], and Yujiang Li [b,*]

[a] *Key Laboratory of Colloid and Interface Science of Education Ministry, Shandong University, Jinan, 250100, PR China*

[b] *Shandong Provincial Research Center for Water Pollution Control, School of Environmental Science and Engineering, Shandong University, Jinan, 250100, PR China*

*Corresponding author. Tel: +86-531-88365437. E-mail: wutao@sdu.edu.cn (Tao Wu)

*Corresponding author. Tel: +86-531-88365922. E-mail: yujiang@sdu.edu.cn (Yujiang Li)

Synthesis of graphene oxide (GO) and magnetite-graphene oxide (MGO)

GO was synthesized using an improved Hummers method with slight modification. First, a mixture of concentrated H_2SO_4 (45 mL) and H_3PO_4 (5 mL) was added to graphite powder (2.0 g), while a magnetic agitation and an ice-bath were maintained for 30 min. $KMnO_4$ (6.0 g) was then slowly added into the mixture, and stirring was continued in the ice-bath for another 30 min. Second, after another 1 h of stirring at 35ºC, DI water (98 mL) was continuously dropped into the mixture while the stirring was maintained. The reaction was then warmed to 98ºC and stirred for another 30 min. Then, a solution mixed with H_2O_2 (5%, 15 mL), HCl (5%, 20 mL), and DI water (280 mL) was further added into the yellow suspension with stirring at 60ºC for 30 min. The graphite oxide particles were separated from the excess liquid by decantation after static settlement for 2 h. The obtained pate-like material was centrifugally cleaned repeatedly using DI water at 8000 rpm until neutral pH. The resulting graphite oxide precipitate was subsequently exfoliated by ultrasonication at room temperature after adding DI water (500 mL) to attain GO dispersion (5 g/L). The GO dispersion was then freeze-dried, and a fine golden yellow powder of GO was achieved.

MGO composites were synthesized by co-precipitating iron salts onto GO nanosheets in basic solution. GO dispersion (400 mL) was placed in a 500 mL round-bottom flask and heated to remove dissolved oxygen at 100ºC. $FeCl_3 \cdot 6H_2O$ (0.9328 g) and $FeSO_4 \cdot 7H_2O$ (0.5992 g) were added into the dispersion while a magnetic agitation was maintained at 90ºC under N_2 protection for 30 min. $NH_3 \cdot H_2O$ solution (25%, 15 mL) was added, and the mixture was continually stirred at 90ºC under N_2 protection for

an additional 30 min. The resulting black precipitate was magnetically separated, and MGO dispersion was obtained by washing thoroughly with DI water to neutral pH. The final product, MGO powder, was obtained by a freeze-drying process.

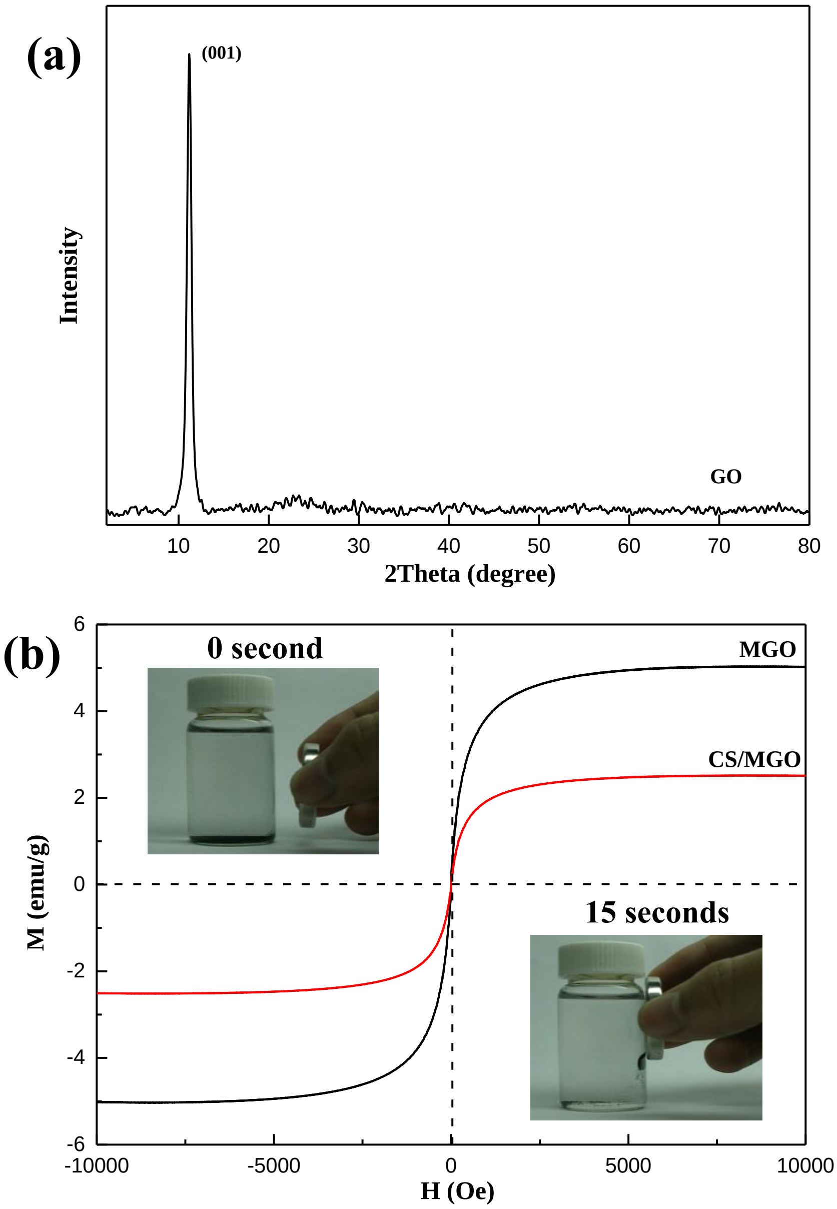

Supplementary Figure S1. XRD pattern of GO (a); magnetization curves of MGO and CS/MGO (b). And the photos of magnetic seperation are presented with the top left and bottom right insert.

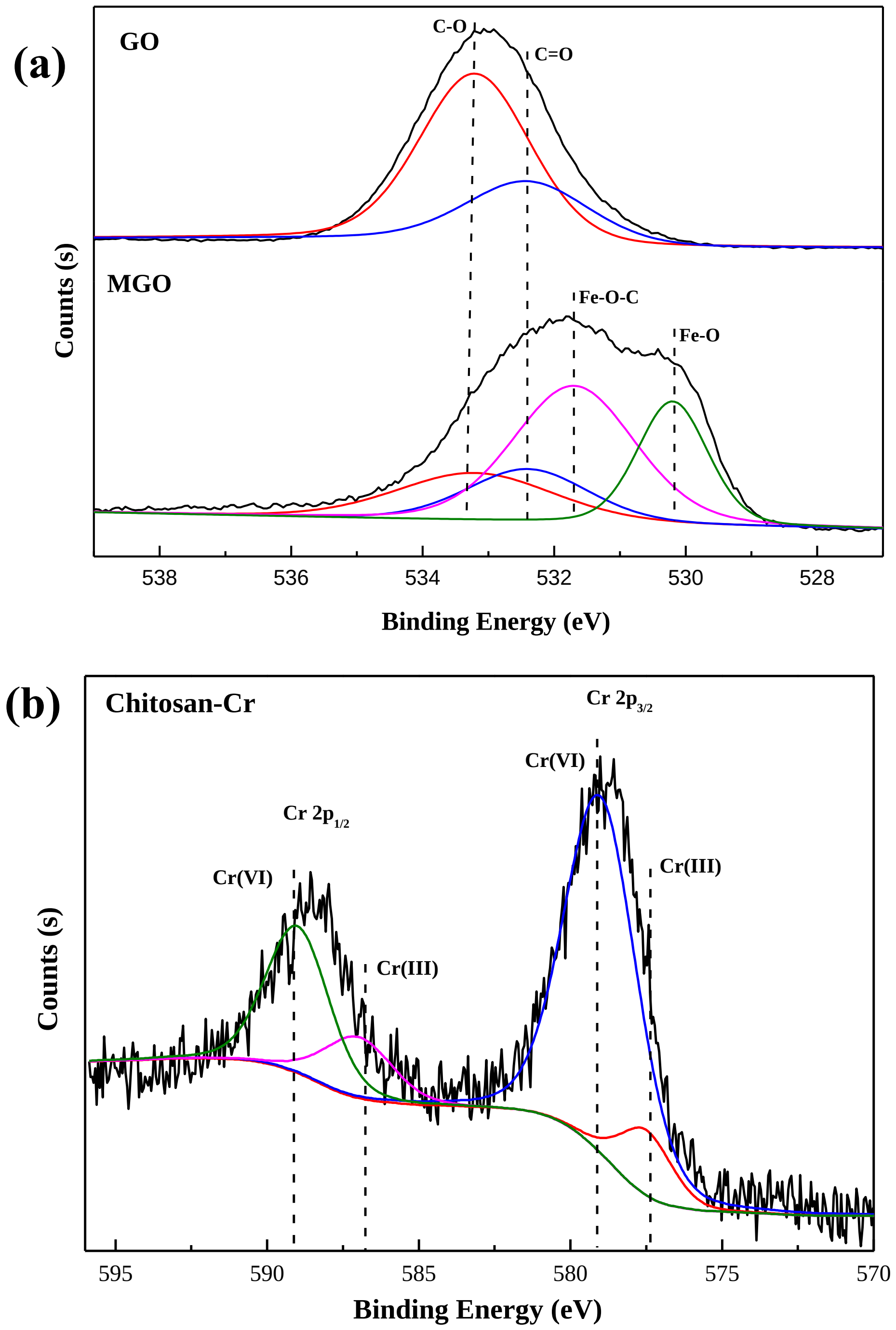

Supplementary Figure S2. XPS O 1s spectra of GO and MGO (a); XPS Cr 2p spectra of chitosan-Cr (b).

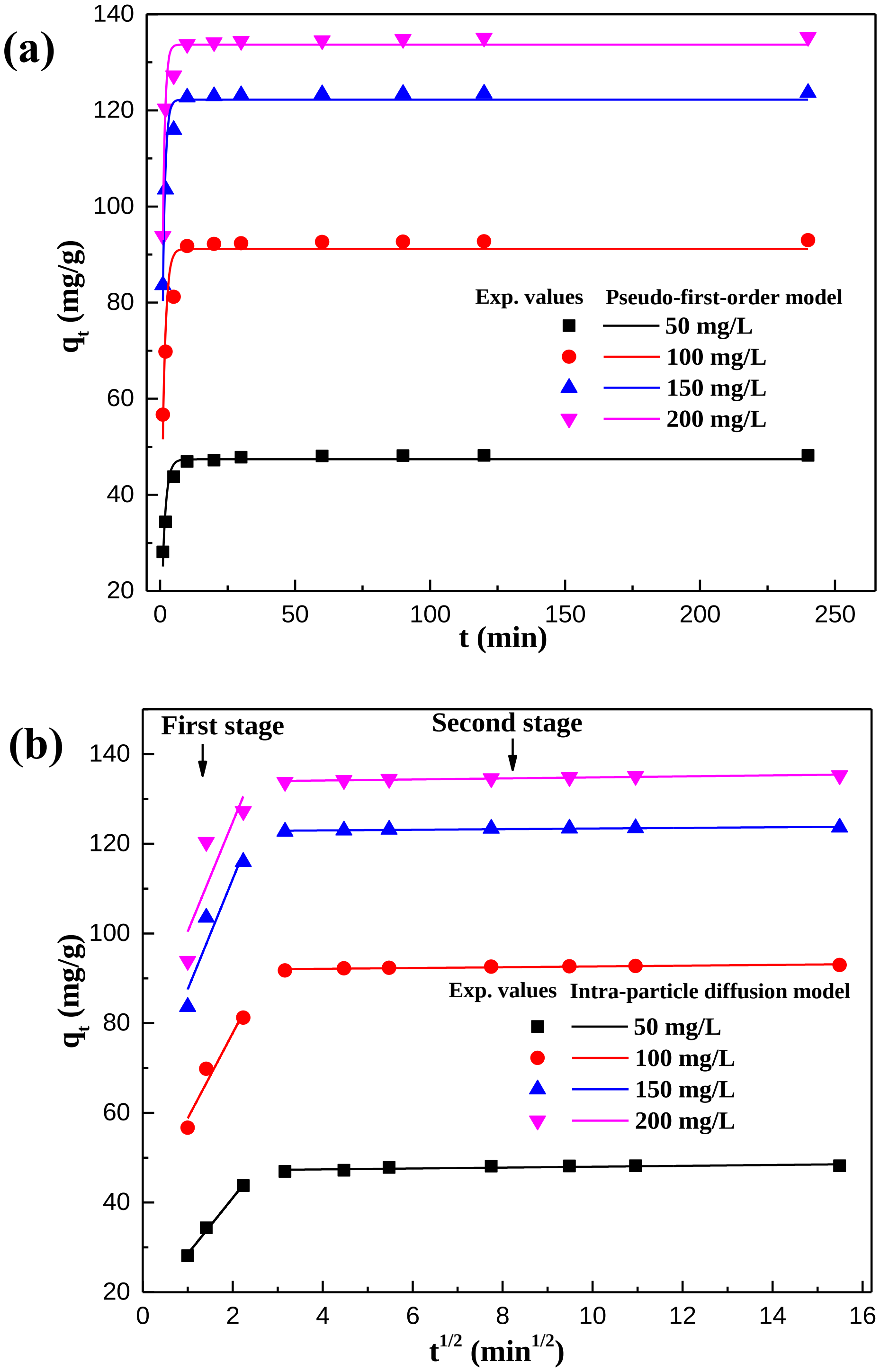
(a)
140
120
100
80
60
40
20
0
50
100
150
200
250
t (min)
q$_t$ (mg/g)
Exp. values
Pseudo-first-order model
50 mg/L
100 mg/L
150 mg/L
200 mg/L
(b)
First stage
Second stage
140
120
100
80
60
40
20
0
2
4
6
8
10
12
14
16
t$^{1/2}$ (min$^{1/2}$)
q$_t$ (mg/g)
Exp. values
Intra-particle diffusion model
50 mg/L
100 mg/L
150 mg/L
200 mg/L

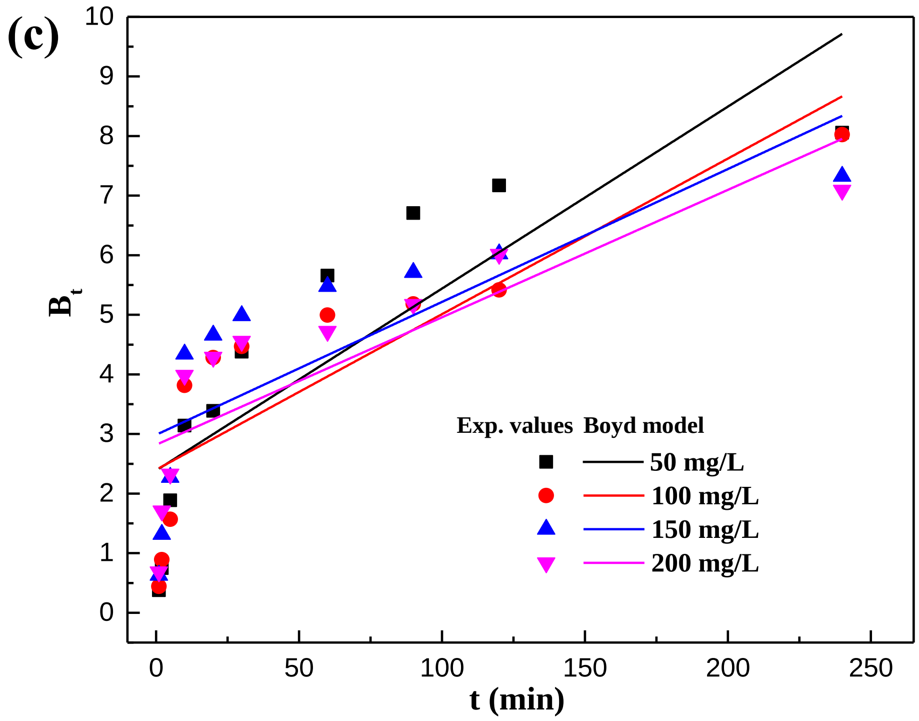

Supplementary Figure S3. Fitting of pseudo-first-order (a), intra-particle diffusion (b), and Boyd (c) kinetic models for the adsorption of Cr(VI) onto CS/MGO composites.

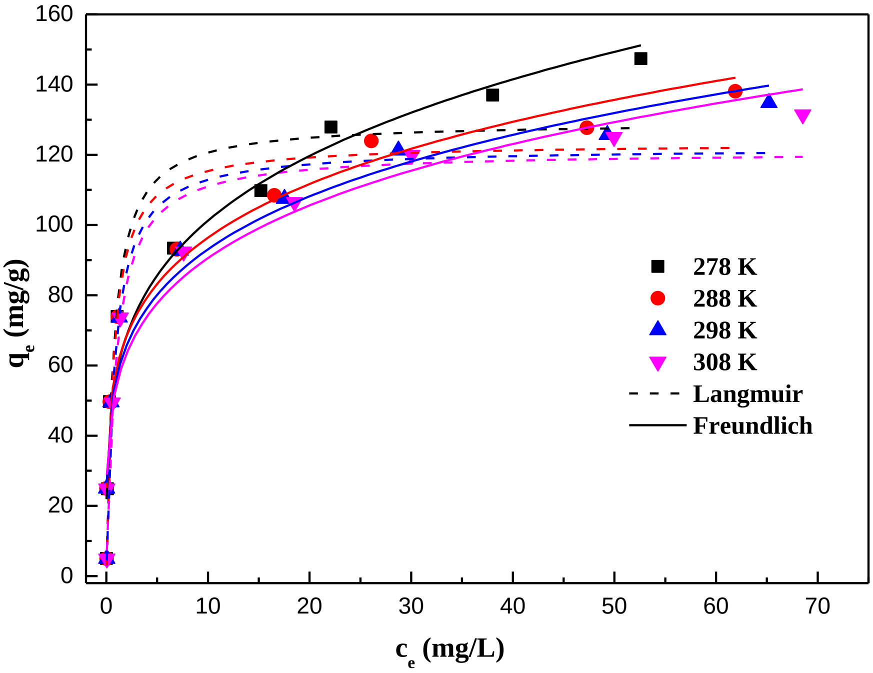

Supplementary Figure S4. Adsorption isotherm of Cr(VI) on CS/MGO composites and fitting of Langmuir and Freundlich model to the experimental values.

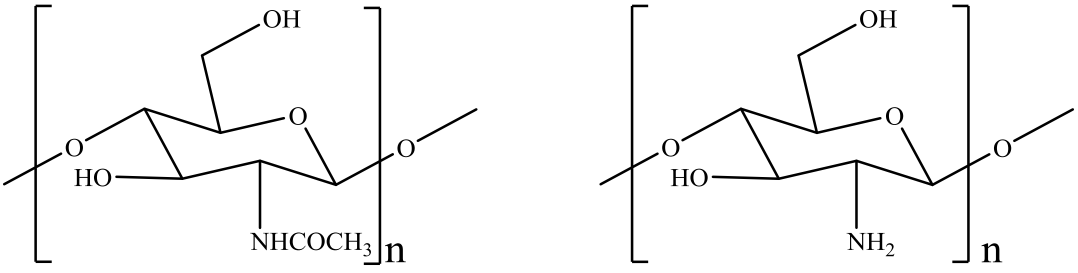

(a) (b)

Supplementary Figure S5. Chemical structure of chitin (a) and chitosan (b).

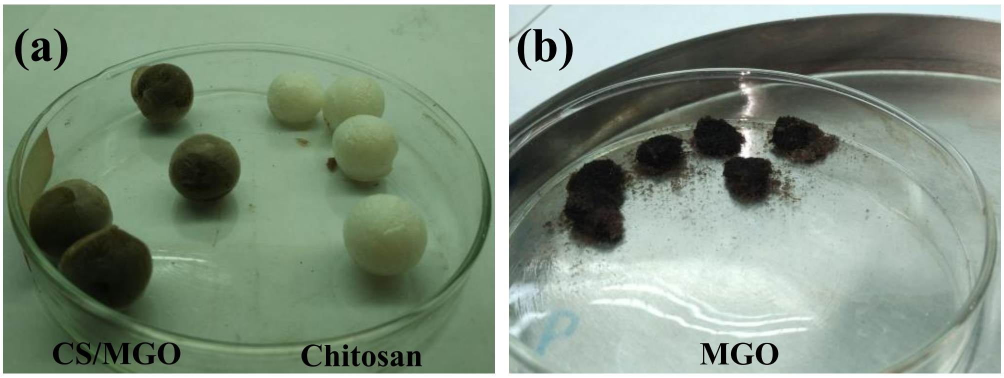

Supplementary Figure S6. Photographs of CS/MGO beads (a, left), chitosan beads (a, right), and MGO powder (b).

Supplementary Table S1. The content of chemical bonds in CS/MGO and CS/MGO-Cr from XPS data.

Sample	CS/MGO	CS/MGO-Cr
C-C, %	36.40	13.13
C-N, %	17.80	25.60
C-O, %	32.72	30.81
C=O, %	10.13	19.50
O-C=O, %	2.95	10.96
$-NH_2$, %	71.54	25.72
O=C-NH-, %	13.26	30.44
$-NH_3^+$, %	15.20	43.84

Supplementary Table S2. The content of Cr(III) and Cr(VI) in chitosan-Cr, CS/GO-Cr, and CS/MGO-Cr from XPS data.

Sample	Chitosan-Cr	CS/GO-Cr	CS/MGO-Cr
Cr(III), Cr $2p_{3/2}$, %	21.71	73.63	86.74
Cr(VI), Cr $2p_{3/2}$, %	78.29	26.37	13.26
Cr(III), Cr $2p_{1/2}$, %	31.00	75.26	89.42
Cr(VI), Cr $2p_{1/2}$, %	69.00	24.74	10.58

Supplementary Table S3. The final pH after adsorption of Cr in aqueous solution.

Initial pH	Final pH
1.03	1.08
2.04	2.15
3.00	3.55
4.01	4.67
5.02	5.37
6.01	6.57
7.02	7.66
8.03	8.23
9.01	8.42
10.00	9.97
11.01	10.92
12.01	11.87
